# Prophylactic mesh reinforcement of stomas: a cost-effectiveness meta-analysis of randomised controlled trials

**DOI:** 10.1007/s10151-018-1774-5

**Published:** 2018-05-07

**Authors:** J. M. Findlay, C. P. J. Wood, C. Cunningham

**Affiliations:** 1 Oxford Colorectal Centre, Churchill Hospital, Oxford University Hospitals NHS Foundation Trust, Oxford, UK; 2 Department of Oncology, University of Oxford, Old Road Campus Research Building, Oxford, OX3 7DQ UK

**Keywords:** Parastomal hernia, Surgical mesh, Stoma, Meta-analysis

## Abstract

**Background:**

Previous meta-analyses of randomised controlled trials (RCTs) have suggested a reduction in parastomal hernias (PSH) with prophylactic mesh. However, concerns persist regarding variably supportive evidence and cost. We performed an updated systematic review and meta-analysis to inform a novel cost-effectiveness analysis.

**Methods:**

The PubMed, EMBASE and Cochrane Centre Register of Controlled Trials databases were searched (February 2018). We included RCTs assessing mesh reinforcement during stoma formation. We assessed PSH rates, subsequent repair, complications and operative time. Odds ratios (OR) and numbers needed to treat (NNT) were generated on intention to treat (ITT) and per protocol (PP) bases. These then informed cost analysis using 2017 UK/USA reimbursement rates and stoma care costs.

**Results:**

Eleven RCTs were included. Four hundred fifty-three patients were randomised to mesh (PP 412), with 454 controls (PP 413). Six studies used synthetic meshes, three composite and two biological (91.7% colostomies; 3.64% ileostomies, 4.63% not specified). Reductions were seen in the number of hernias detected clinically and on computed tomography scan. For the former, ITT OR was 0.23 (95% confidence interval 0.11–0.51; *p* = 0.0003; *n* = 11); NNT 4.17 (2.56–10.0), with fewer subsequent repairs: OR 0.29 (0.13–0.64; *p* = 0.002; *n* = 7; NNT16.7 (10.0–33.3). Reductions persisted for synthetic and composite meshes. Operative time was similar, with zero incidence of mesh infection/fistulation, and fewer peristomal complications. Synthetic mesh demonstrated a favourable cost profile, with composite approximately cost neutral, and biological incurring net costs.

**Conclusions:**

Reinforcing elective stomas with mesh (primarily synthetic) reduces subsequent PSH rates, complications, repairs and saves money. We recommend that future RCTs compare mesh subtypes, techniques, and applicability to emergency stomas.

## Introduction

Parastomal hernias (PSH) remain common, occurring in 6% of patients with loop ileostomies and almost half with end colostomies, and can profoundly affect quality of life, impair productivity and generate substantial healthcare costs [1].

Mesh PSH repair was originally described in 1997 [1] and is generally accepted as the most effective method. However, a number of recent randomised controlled trials (RCTs) have variably suggested that reinforcing stomas prophylactically with mesh reduces the risk of PSH. Consequent systematic reviews and meta-analyses have supported this position [2], and also variably suggested risk of complications to be minimal, addressing one of two major reservations towards widespread acceptance and change in practice, beyond heterogeneity of the evidence base. However, none have addressed the second: financial cost. We aimed to perform an updated systematic review and meta-analysis, informing a cost-effectiveness analysis.

## Materials and methods

### Literature search

We searched the PubMed, EMBASE and Cochrane Centre Register of Controlled Trials databases up to 9 February 2018, in accordance with the Preferred Reporting Items for Systematic Reviews and Meta-Analyses and Meta-analysis Of Observational Studies in Epidemiology guidelines using the following search term: (mesh OR hernia) AND (stoma OR parastomal OR colostomy OR ileostomy) AND (randomised OR controlled OR trial). Bibliographies of retrieved articles were searched. Two authors (JMF and CPJW) screened articles independently.

### Inclusion and exclusion criteria

We included English language RCTs randomising patients undergoing stoma formation (loop/end ileostomy/colostomy) to mesh reinforcement (synthetic/composite/biological) or not.

### Endpoints

Primary outcomes were development of PSH (clinically and/or radiologically detected, as defined in individual studies) and subsequent repair. These were then used to inform a cost-effectiveness analysis. Our secondary outcomes were complications (as defined by individual studies) and operating time [3].

### Data collection

Data were extracted independently (JMF and CPJW). We contacted one study’s authors to clarify ITT and PP results [4].

### Assessment of bias and evidence quality

Bias was assessed using the Cochrane Collaboration ‘Risk of bias’ tool [5], with recommendations stratified using GRADE [6].

### Meta-analysis and other statistical analysis

Meta-analysis was performed using RevMan v5.2 (The Cochrane Collaboration); additional analyses using R v3.02 (R Core Team). Heterogeneity was quantified by *I*^2^ and Chi-square. Odds ratios (OR), absolute risk reduction (ARR) and 95% confidence intervals (CI) were calculated used random effects when *I*^2^ > 50%. Both intention to treat (ITT; patients randomised to treatment arms, irrespective of subsequent protocol violations), and per protocol (PP; only patients receiving treatment and follow-up as stipulated) analyses were performed. Funnel plots were inspected for asymmetry. Perceived publication bias was corrected using the ‘trim and fill’ method [7]. *p* < 0.05 was considered significant. Sensitivity analyses were conducted.

### Cost-effectiveness analysis

We performed a two-tiered analysis. The first (most conservative) comprised the cost of elective PSH repair and mesh. Net inpatient PSH repair costs were obtained from 2017 United States of America (USA) Medicare national average reimbursement rates, and United Kingdom (UK) National Health Service tariff, (USD$7572/$9989/$17,143; and GBP£1775/£2646/£4328 for no, intermediate and major comorbidities, respectively; procedure codes MS-DRG Codes 353-355). Mesh costs were obtained from Medtronic, Ethicon and Elemental Healthcare for representative 10 × 15 cm synthetic, composite and biological meshes. Mean costs were: £32.77/$42.60, £296.64/$385.64 and £1650/$2145, respectively. A cost range was calculated based on cheapest and most expensive meshes and tariffs.

The second tier included additive stoma care for patients with a PSH per year for 2 years (typical follow-up of included RCTs). This was estimated by assuming PSH resulted in 25% more frequent leakage with a commensurate increase in cost of appliances and care (£3024.80–4315/$3932.5–5609.50) [8, 9,10].

## Results

### Literature search

**Fig. 1:**
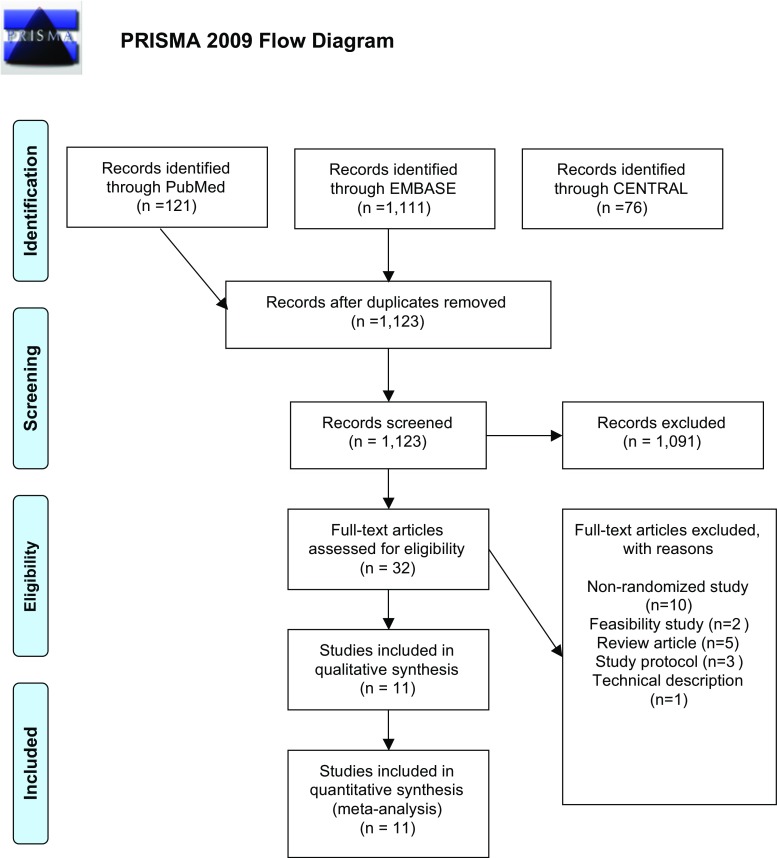
Preferred reporting items for systematic reviews and meta-analyses (PRISMA) diagram

One thousand one hundred twenty-three studies were identified; 32 full texts were appraised; 11 RCTs were included (Fig. 1). Four hundred fifty-three patients were randomised to mesh (per protocol (PP) 412), with 454 controls (PP 413). Six studies used synthetic mesh [3, 4, 11–14], three composite [15–17] and two biological [18, 19]. Eight studies only included end colostomy, one only loop ileostomy [19], either ileostomy or colostomy [18], and 1 unspecified stoma formation [14] Overall, 33 patients (PP; 3.64%) had ileostomy formation, and 832 (91.7%) colostomy [42 (4.63%) not specified]. One hundred eighty-three patients (21.6%) underwent a laparoscopic operation, 523 (61.8%) open [140 (16.5%) not specified]. Five studies included only abdominoperineal excision for rectal/pelvic cancer [12, 13, 15–17]; one end ileostomy/colostomy for low rectal cancer [14]; three laparotomy with end colostomy [3, 4, 11]; one permanent end ileostomy/colostomy for any indication [18]; and one loop ileostomies after major anorectal surgery [19]. All studies included elective patients, other than two (including four and one emergency laparotomies, respectively) [3, 11]. Two studies excluded patients with a body mass index > 35 [13, 18]. Follow-up varied: up to 12 months in 7 [3, 4, 13, 15–17, 19], 24 months in 2 [14, 18]; 1–4 years [12] and 1–5 years [11].

### Endpoints

In patients randomised to prophylactic mesh, significantly fewer PSH were detected clinically: intention to treat (ITT) OR 0.23 (0.11–0.51; *p* = 0.0003; *n* = 11; Table 1; GRADE recommendation moderate; Table 2). Number need to treat (NNT) was 5.00 (3.22–10.0). PP OR was 0.37 (0.26–0.53). Lesser reductions were seen for CT-detected hernias: OR 0.43 (0.26–0.71; *p* = 0.001; *n* = 9). Subgroup analysis demonstrated significance for synthetic and composite meshes, and end colostomies. PP results were similar.

**Table 1:** Effects on clinically detected hernias and subsequent repair

Analysis	OR	95% CI	*p*	*n* studies	*I*^2^* (%)	Effects model	Adjusted for publication bias?
Clinically detected hernias							
*All meshes*							
ITT	0.23	0.11–0.51	0.0003	9	66	Random	Yes
PP	0.37	0.26–0.53	0.0008	9	67	Random	Yes
*Synthetic mesh*							
ITT	0.11	0.05–0.22	< 0.0001	6	81	Random	No
PP	0.19	0.05–0.71	0.001	6	81	Random	No
*Biological mesh*							
ITT	0.59	0.21–1.64	0.310	2	37	Fixed	No
PP	0.41	0.14–1.27	0.130	2	6	Fixed	No
*End colostomy*							
ITT	0.20	0.07–0.58	0.0001	6	80	Random	No
PP	0.21	0.07–0.69	< 0.0001	6	81	Random	No
*CT-detected hernias*							
ITT	0.43	0.26–0.71	0.0010	8	49	Random	Yes
PP	0.40	0.20–0.77	0.0060	8	62	Random	Yes
Parastomal hernia repair							
*All meshes*							
ITT	0.37	0.24–0.55	< 0.0001	7	32	Fixed	Yes
PP	0.33	0.16–0.68	0.003	7	62	Random	Yes
*Synthetic*							
ITT	0.16	0.04–0.55	< 0.0001	5	81	Random	No
PP	0.15	0.04–0.57	0.006	4	0	Fixed	No
Composite							
ITT	0.55	0.13–2.36	0.420	3	0	Fixed	No
PP	0.57	0.13–2.42	0.440	3	0	Random	No
Biological							
ITT	0.47	0.11–1.99	0.310	1	NA	Fixed	No
PP	0.46	0.09–2.44	0.360	1	NA	Fixed	No
*End colostomy*							
ITT	0.27	0.10–0.75	0.0100	6	0	Fixed	No
PP	0.28	0.10–0.80	0.020	6	0	Fixed	No

*OR* odds ratio, *CI* confidence interval, *CT* computed tomography, *ITT* intention to treat, *PP* per protocol

*Before adjusting for publication bias

**Table 2:** GRADE recommendations

Analysis	LOE	Risk of bias	Inconsistency	Indirectness	Imprecision	Publication bias	Quality
*Clinically detected hernias*							
All	RCT (+4)	Serious (−1)	Consistent (+1)	None	None	Detected (−1)	Moderate
Synthetic	RCT (+4)	Serious (−1)	None	None	None	Not detected	Moderate
Biological	RCT (+4)	Very serious (−2)	None	Serious (−1)	Serious (−1)	Not detected	Very low
*CT-detected hernias*							
All	RCT (+4)	Serious (−1)	Consistent (+1)	None	None	Detected (−1)	Moderate
Synthetic	RCT (+4)	Serious (−1)	None	None	None	Not detected	Moderate
Composite	RCT (+4)	Serious (−1)	None	None	None	Not detected	Moderate
*Complications*							
All	RCT (+4)	Very serious (−2)	None	None	Serious (−1)	Detected (−1)	Very low
Synthetic	RCT (+4)	Very serious (−2)	None	None	Serious (−1)	Not detected	Very low
Composite	RCT (+4)	Very serious (−2)	None	None	Serious (−1)	Not detected	Very low
*Hernia repair*							
All	RCT	Very serious (−2)	None	None	None	Not detected	Low
*Operative time*							
ITT	RCT	Very serious (−2)	None	Serious (−1)	Serious (−1)	Not detected	Low

*RCT* randomised controlled trial, *LOE* level of evidence, *CT* computed tomography

This translated into fewer subsequent repairs: OR 0.29 (0.13–0.64; *p* = 0.002); NNT 16.7 (10.0–33.3); GRADE recommendation low, persisting for synthetic mesh: NNT 11.1 (7.14–25.0). There was zero incidence of mesh infection/fistulation. There were fewer minor peristomal complications with mesh (GRADE recommendation very low): ITT OR 0.48 (0.30–0.77), *p* = 0.002; *n* = 9. Operative time was similar: mean difference 5.16 min (−13.4–23.8; *p* = 0.590; *n* = 6).

### Evidence quality

Overall, quality ranged from very low to moderate (Table 2). The evidence for PSH reductions was moderate (further research being unlikely to change the effect direction, but likely to change the estimate). This was affected by serious risk of bias and probable publication bias, but enhanced by consistency of effect. Evidence for biological meshes was very low, indicating high uncertainty (very serious bias, indirectness including use of mesh for temporary loop ileostomies, and imprecision in effect sizes). Evidence for the reductions in repairs were low, limited mainly by very serious potential bias (inevitable variability in thresholds for repair), indicating that further research may affect direction of effect. Quality for complication reductions was very low (very serious risk of bias, particularly in complication reporting and definitions, and imprecision in effect sizes).

### Cost analysis

Routine use of synthetic mesh would save money (Table 3). Considering only operative costs, in the USA overall cost per patient was USD$ −622.36 to −1513.21 (GBP£ +135.91 to −365.91) Including stoma costs, this was reduced further. Composite meshes were roughly cost neutral when considering operative costs, with lesser savings when considering stoma costs. The expense of biological mesh resulted in net costs.

**Table 3:** Cost-effectiveness

Mesh	NNT	Net cost per patient	
		Lowest	Highest
USD $			
Synthetic	11.1	−1513.21	−622.36
Composite	33.3	−278.80 −	+468.00
Biological	20	+792.85	+2351.40
GBP £			
Synthetic	11.1	−365.91 − −	+135.91 –
Composite	33.3	+ 106.03 − −	+306.70
Biological	20	+ 983.6 −	+2011.25
*Plus additive stoma costs*			
USD $			
Synthetic	3.45	−2138.58	−1192.29
Composite	6.68	−698.68	+173.65
Biological	16.67	+624.60	+2233.45
GBP £			
Synthetic	3.45	−991.27	−552.32
Composite	6.68	−216.95	+80.27
Biological	16.67	+854.18	+1920.52

*NNT* number needed to treat, *USD* US dollar, *GBP* British pound sterling

## Discussion

In the most up-to-date meta-analysis of 11 RCTs involving 907 patients, we found reinforcing elective stomas with prophylactic mesh reduced the incidence of subsequent PSH and repair. There was no significant increase in operative time, with substantial cost savings for synthetic meshes, and fewer peristomal complications. The evidence was less clear (and of lower quality) for composite and biological meshes, with the extra cost of the latter seeming to subsume potential savings.

These findings agree with recent meta-analyses [2]. However, none have assessed costs, anecdotally perceived to be significant and hence a barrier to changes in practice. As such we believe our findings are novel, and provide context to the evolving evidence base. This notwithstanding, we acknowledge a number of limitations, beyond those of constituent study heterogeneity, bias and methodology. We used GRADE recommendations to provide context, but we were unable to perform meaningful meta-regression on the basis of individual study quality. Similarly, we were unable to compare stoma types, other than analysis of end colostomy.

Ours is also the first meta-analysis to include the recent publication by Odensten et al. [3]. This was notable both as the largest study to date, with relatively little bias and its finding of a lack of effect. The reasons for this are unclear; however, the rates of PSH in both arms of the trial were high, at approximately 30%.

Our cost analysis was by necessity constrained by the very limited data available regarding cost to patients, and projections by length of study follow-up. It is very likely that our estimates are conservative: NNT would likely reduce further with time, and we could not reliably account for significant additional costs, including the economic costs to patients who develop hernias (for example time off work) beyond stoma care. Furthermore, it is likely that commensurate benefits in quality of life would be seen beyond the financial, but these areas have received little attention in the literature.

We recommend that these be explored in the next generation of RCTs and furthermore, that these compare mesh types and reinforcement approach. However, perhaps most pressing is whether reinforcement is of benefit in patients undergoing emergency stomas, particularly a Hartmann’s procedure. A substantial proportion of these patients never undergo reversal, whilst they are perhaps most vulnerable to the consequences of PSH.

## Conclusions

We found that reinforcing stomas with mesh reduces subsequent PSH rates, repair and complications, and should be considered routinely. Synthetic mesh has the best evidence profile and results in potentially substantial cost savings. The additional cost of composite and biological mesh does not yet appear to confer any additional benefit.
